# Traditional method of rhubarb processing optimized by combining flavor analysis with anthraquinone content determination

**DOI:** 10.3389/fnut.2024.1406430

**Published:** 2024-06-12

**Authors:** Taotao Liu, Miao Yu, Yue Dai, Yongqing Xiao, Li Li

**Affiliations:** Institute of Chinese Materia Medica, China Academy of Chinese Medical Sciences, Beijing, China

**Keywords:** electronic tongue, gas chromatography-ion mobility spectrometry, rhubarb, taste flavor, high-performance liquid chromatography

## Abstract

**Introduction:**

Rhubarb is a popular food that relieves constipation and aids with weight loss. The traditional method of preparation, includes steaming and sun-drying rhubarb nine times (SDR-9) to reduce its toxicity and increase efficacy.

**Methods:**

Flavor analysis includes odor analysis by gas chromatography–ion mobility spectrometry and taste characterization using an electronic tongue.

**Results:**

Odor analysis of the samples prepared through SDR-9 identified 61 volatile compounds, including aldehydes, esters, alcohols, ketones, acids, alkenes, and furans. Of these, 13 volatile components were the key substances associated with odor. This enabled the process to be divided into two stages: 1–5 times of steaming and sun-drying and 6–9 times. In the second stage, SDR-6 and SDR-9 were grouped together in terms of odor. Analysis using electronic tongue revealed that the most prominent taste was bitterness. A radar map indicated that the bitterness response was the highest for raw rhubarb, whereas that for processed (steamed and sun-dried) rhubarb decreased. Orthogonal partial least squares discriminant analysis (OPLS-DA) clustering results for SDR-6 and SDR-9 samples indicated that their tastes were similar. Anthraquinones were analyzed via high-performance liquid chromatography; moreover, analysis of the taste and components of the SDR samples revealed a significant correlation.

**Discussion:**

These results indicate that there are similarities between SDR-6 and SDR-9 in terms of smell, taste, and composition, indicating that the steaming and sun-drying cycles can be conducted six times instead of nine.

## 1 Introduction

Rhubarb (Rhei Radix et Rhizoma), including *Rheum palmatum* L., *Rheum tanguticum* Maxim. ex Balf, and *Rheum officinale* Baill., has a long history of use as a conventional Chinese medicine worldwide (1). It has a variety of pharmacological properties, including antibacterial, anti-inflammatory, antitumor, hepatoprotective, renoprotective, immunoregulatory, free radical scavenging, purgative, and cardiovascular protective properties (2). Rhubarb can be used clinically to treat obesity as it inhibits proinflammatory signaling pathways and modulates glucose–lipid homeostasis (3). Rhein, a crucial component of rhubarb, can inhibit obesity caused by a high-fat diet, decrease fat mass, and reduce the size of white and brown adipocytes. It can also lower the levels of serum cholesterol, low-density lipoprotein cholesterol, and fasting blood glucose in mice (4). Aloe-emodin, emodin, chrysophanol, and physcion in rhubarb are other important chemicals that promote weight loss (5). Europeans and Americans have regarded edible rhubarb as a palatable food since the 19th century (6); however, long-term consumption of raw and processed rhubarb increases the risk of melanosis coli and liver and kidney damage, which are primarily attributed to anthraquinones (7, 8). Compared with raw rhubarb, the processed version has relatively fewer side effects and rarely causes diarrhea (9).

In China, rhubarb is commonly used after processing. Four processes have been recorded in the Chinese Pharmacopeia, which include cleaning, roasting with wine, steaming, and charcoal frying to obtain raw rhubarb, roasted rhubarb with wine, steamed rhubarb with wine, and carbonized rhubarb, respectively (10). Different methods are used throughout the world for various purposes (11). Steaming and sun-drying nine times is one characteristic method of processing Chinese Materia Medica and is commonly used to increase the activity of most traditional Chinese medicines and reduce or avoid side effects. The long-term consumption of raw rhubarb can damage renal function in rats; however, processed rhubarb reduces the risk of renal injury (9). Forty-three traditional Chinese medicines, including rhubarb, are steamed and dried several times, as described in the ancient medical literature (12). Overall, the process of steaming and sun-drying nine times yields good clinical effects and is worthy of further study. However, the pharmacological activities and primary active ingredients of rhubarb have been the main focus of most studies (6). There are few studies on the odor and taste of rhubarb processed by steaming and sun-drying nine times. Moreover, the processing period is long and tedious, this traditional Chinese medicine is subject to rot and mildew during exposure to the climate. Therefore, it is necessary to devise a method to simplify the preparation process.

Based on the above findings, after steaming and sun-drying nine times, the odor and taste of rhubarb change to varying degrees, which is a vital indicator for process control and quality evaluation. However, as traditional evaluation methods primarily rely on sensory identification by humans and other subjective analyses, the results are inevitably affected by sensory differences and the detection environment. As a result, ensuring the objectivity and accuracy of the evaluation is an important issue for the quality evaluation of traditional Chinese medicines. Artificial intelligence is rapidly developing, and electronic tongues and noses have become widely available in recent years. As a modern, intelligent, sensory, qualitative analysis, and testing tool, the electronic tongue consists of an interactive and sensitive sensor array and a signal acquisition circuit that is combined with a data processing method based on pattern recognition. An artificial lipid bilayer membrane with unique and wide selectivity of an area can be used to directly output taste values, including sour, sweet, bitter, astringent, fresh, salty, bitter aftertaste, and richness from a sample solution (13). It may be applied to studies of food (14), beverages (15), tea (16), alcohol discrimination (17), and environmental analysis (18). It can detect overall taste, but not the specific compounds involved (19).

In addition to the electronic tongue, gas chromatography–ion mobility spectrometry (GC–IMS) is a novel technique that can be used to identify ions based on differences in the migration rates of various gas-phase ions in an electric field. In the ionization region, gas molecules are converted into charged ions, which then enter a drift tube. Identification and analysis are performed based on the different migration velocities of gaseous ions in the electric field. GC–IMS is a powerful analytical method that combines the simplicity and rapidity of GC with the high-resolution and accurate analysis of IMS. The advantages of this method are its low detection limit, short analysis time, and ease of operation (20). GC–IMS is increasingly being used for analyzing food flavors (21), discriminating traditional medicines (22, 23), and classifying white wines (24).

The flavor index is a traditional method of evaluating the quality of rhubarb. The effective component is not only the common index of rhubarb quality but also the standard component of quality control in Chinese Pharmacopeia. Thus, it can be used as the standard of evaluation. To simplify the process and improve efficiency, the flavor and components of rhubarb prepared by steaming and sun-drying nine times were analyzed using GC–IMS, the electronic tongue, and high-performance liquid chromatography (HPLC).

## 2 Materials and methods

### 2.1 Rhubarb processing and sample preparation

Fresh *Rheum palmatum* L. was cleaned and prepared based on the Chinese Pharmacopeia (2020 edition) (The State Pharmacopeia Committee of the People’s Republic of China, 2020) to obtain raw rhubarb samples (Shengpian in Chinese). Initially, 2.5 kg of Huangjiu was added to 5.0 kg of Shengpian and mixed well until fully absorbed. Using an induction cooker (1800 w), the raw rhubarb was steamed for 4 h, followed by natural sun-drying. A 0.5-kg sample was used in one cycle of steaming and sun-drying (hereinafter referred to as SDR-1); the remaining sample was repeatedly subjected to the same process, with 0.5-kg sample removed after each cycle. A total of nine samples (SDR-1–9) were obtained and each sample was prepared in triplicate. Finally, the samples were powdered using 40-mesh sieve and a grinder.

### 2.2 GC–IMS analysis

The FlavourSpec flavor analyzer (G.A.S., Dortmund, Germany) uses GC–IMS technology to measure the volatile headspace components. Rhubarb samples (3.0 g) were incubated in a 20-mL headspace vial for 20 min at 60°C. A 200-μL sample was then injected into an MXT-WAX metal capillary GC column (30 m × 0.53 mm; Restek Corporation, the United States) at 85°C with nitrogen (99.99%) as the carrier gas. Flow rates started at 2 mL/min for 2 min and increased to 10 mL/min for 8 min, then to 100 mL/min for 10 min, and finally to 100 mL/min for 20 min. The sample then entered the ion transfer tube. After the molecules were ionized in the ionization region, they migrated to the Faraday disk for detection by an electric field and reverse drift gas to achieve separation. Nitrogen (99.99%) was used as the drift gas with a flow rate of 150 mL/min. The GC–IMS instrument with software (G.A.S.) was used to obtain the three- and two-dimensional spectra, fingerprints, and principal component analysis (PCA) graph.

### 2.3 Quantitative analysis of anthraquinones

#### 2.3.1 Reagents and materials

The nine reference components are chrysophanol-8-O-β-D-glucoside, emodin-8-O-β-D-glucoside, aloe-emodin-3-(hydroxymethyl)-O-β-D-glucoside, physcion-8-O-β-D-glucoside, aloe-emodin, rhein, emodin, chrysophanol, and physcion (designated A–I), which were purchased from the National Institutes for Food and Drug Control. The purity of all components was greater than 98%. HPLC-grade solutions, methanol, and reagents were purchased from Thermo Fisher Scientific (United States).

#### 2.3.2 Chromatographic conditions

The HPLC system (Waters, United States) consisted of a Waters 2695 Separations Module and Waters 2998 PDA detector. The output signal of the detector was recorded using an Empower 3 workstation. For the separation of the sample, a Roc C18 column (4.6 mm × 250 mm, 5 μm) was used and the UV detection wavelength was set to 280 and 430 nm. The mobile phase consisted of methanol (A) and 0.1% glacial acetic acid (B) with gradient elution (0–5 min, A 54%; 5–15 min, A 54–64%; 15–20 min, A 64–73%; 20–23 min, A 73–83%; 23–26 min, A 83–90%; 26–27 min, A 90–100%; and 27–33 min, A 100%) at a flow rate of 1.0 mL/min. The injection volume was 10 μL and the column temperature was maintained at 35°C.

#### 2.3.3 Preparation of rhubarb test solution

Raw rhubarb (0.5 g) was placed in a cone bottle with a plug and 25 mL of methanol was added. Ultrasonic extraction lasted for 10 min. After cooling, the solution was filtered through a paper filter prior to HPLC analysis. The sample solution was filtered through a 0.22-μm filter.

#### 2.3.4 Preparation of standard solution

Each standard stock solution comprised nine components, including chrysophanol-8-O-β-D-glucoside (0.0164 mg/mL), emodin-8-O-β-D-glucoside (0.0201 mg/mL), aloe-emodin-3-(hydroxymethyl)-O-β-D-glucoside (0.0097 mg/mL), emodin-8-O-β-D-glucoside (0.0142 mg/mL), aloe-emodin (0.0200 mg/mL), rhein (0.0270 mg/mL), emodin (0.0780 mg/mL), chrysophanol (0.0344 mg/mL), and physcion (0.0222 mg/mL), and was prepared by dissolving in methanol. Six different volumes (1, 5, 10, 15, 20, and 25 μL) of the standard solution were used to establish a calibration curve. A working solution was prepared for each standard. The stock solutions were stored at 4°C.

### 2.4 Electronic tongue analysis

A TS-5000Z electronic tongue (INSET Inc., Japan) was used to analyze the taste characteristics of rhubarb. An artificial lipid membrane sensor in the electronic tongue simulates the taste perception mechanism of living organisms and evaluates taste by detecting changes in membrane potential generated by the interaction between substances and artificial lipid membranes. The five detective sensors include AAE, CT0, CA0, C00, and AE1, which represent umami (richness), saltiness, sourness, bitterness (aftertaste-A), and astringency (aftertaste-B). The first taste and aftertaste corresponding to each sensor and the specific ingredients that impart these tastes are listed in Table 1. Briefly, 100 mL of pure water was added to 1 g of rhubarb and placed in an ultrasonic cleaning machine for 30 min, followed by centrifugation at 3,000 rpm for 5 min. The supernatant was filtered through filter paper, and the filtrate was tested. Each sample was processed three times. Finally, the detection data were transformed into taste values using the electronic tongue software (the Taste Sensing System and Taste Analyzed System applications).

**Table 1:** First taste and aftertaste corresponding to each sensor and the specific ingredients that impart these tastes.

Sensor	Corresponding sense of taste	
	First taste	Aftertaste
AAE	Umami (caused by amino acids and nucleic acids)	Richness (sustainable perception)
CT0	Saltiness (caused by inorganic salts such as table salt)	——
CA0	Sourness (caused by acetic acid, citric acid, and tartaric acid)	——
C00	Bitterness (caused by bitter substances that are perceived as rich at low concentrations)	Aftertaste-B (caused by drinks such as beer and coffee)
AE1	Astringency (caused by astringent substances, perceived as an irritating aftertaste at low concentrations)	Aftertaste-A (caused by drinks such as tea and red wine)
AN0	——	B-bitterness2 (caused by bitter medicine)
BT0		H-bitterness (caused by hydrochloride compounds)

### 2.5 Statistical analysis

The VOCal software and three plug-ins supported by GC–IMS were used to analyze the rhubarb samples. The spectrum of qualitative and quantitative analyses and data may be viewed using VOCal software. The three-dimensional spectrum, two-dimensional top view, and difference spectrum were used to directly compare the spectral differences among samples using the Reporter plug-in. The differences in volatile headspace components were compared using the Gallery Plot plug-in visually and quantitatively using fingerprint. GC–IMS was used to detect the peak intensity response value and the Dynamic PCA plug-in was used to analyze the data. A one-way ANOVA for multiple comparisons was performed using GraphPad Prism software (v.6.02, GraphPad Software, San Diego, CA, United States). To determine the correlation of volatile compounds in rhubarb samples for different steaming and sun-drying times, a correction heatmap was generated using the OriginPro 2021 software (v.9.8.0, OriginLab, Northampton, MA, United States). IBM SPSS Statistics software (R26.0.0.0, IBM, United States) was used for Pearson’s correlation analysis. The data obtained from the electronic tongue was analyzed using the Taste Sensing System (v.2.0.0.0, Insent, Japan) and Taste Analyzed System (v.1.0.0.5, Insent) application. Orthogonal partial least squares discriminant analysis (OPLS-DA) of the electronic tongue data was viewed using SIMCA (v.14.1, Umetrics, Sweden).

## 3 Results

### 3.1 Odor analysis using GC–IMS

#### 3.1.1 Visual topographic plots of rhubarb samples subjected to different steaming and sun-drying times

**Figure 1:**
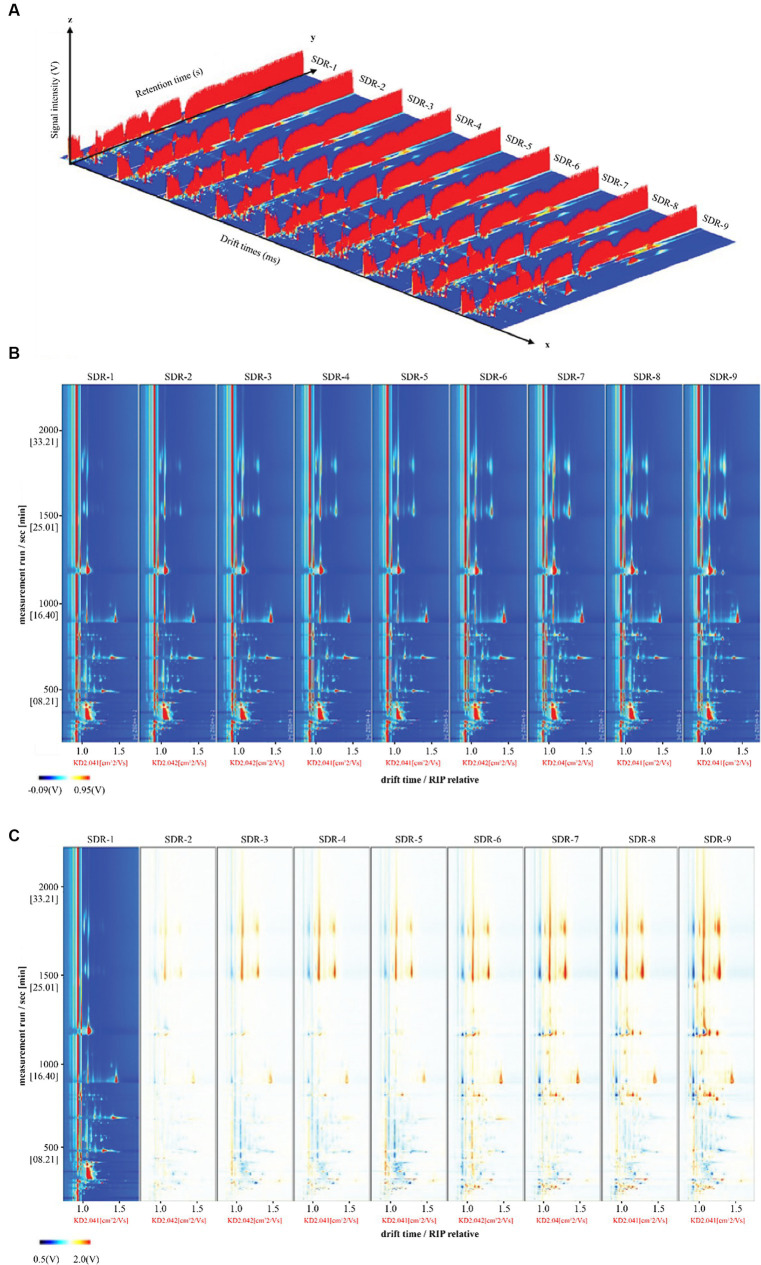
Volatile compounds in rhubarb samples at different steaming and sun-drying times. **(A)** Three-dimensional topography. **(B)** Topographic plot of GC–IMS spectra. **(C)** Comparison of the results from the spectral diagram (SDR-1) with one sample selected as the reference.

The three-dimensional spectrum in Figure 1A presents the differences in the volatile headspace components of the rhubarb samples. The two-dimensional spectrum is presented in Figure 1B. The normalized reaction ion peak occurs at abscissa 1.0, and each point on both sides represents a volatile organic compound. Obvious differences were observed in the volatile organic compounds at different steaming and sun-drying times. SDR-1 was used as the reference sample, and the spectra of the other samples were subtracted from the reference. As indicated in Figure 1C, the background after deduction is white when the two volatile organic compounds (VOCs) are consistent, whereas red and blue backgrounds indicate that the concentrations are higher and lower than those of the reference, respectively. Figure 1C indicates an increase in the content of some volatile components as the number of processing cycles increases. Decreases in the levels of these compounds were also observed.

#### 3.1.2 Volatile compounds in rhubarb samples subjected to different steaming and sun-drying times

A total of 61 VOCs were identified in the rhubarb samples, including aldehydes, alcohols, ketones, esters, and furans, as previously described (25). Among them, 14 aldehydes, 12 alcohols, 12 ketones, and nine esters were the main VOCs. The details of these VOCs are listed in Table 2.

**Table 2:** Gas chromatography–ion mobility spectrometry global area set integration parameters obtained from the rhubarb samples after different cycles of steaming and sun-drying.

Count	Compound	CAS#	Formula	MW	RI	Rt [s]	Dt [a.u.]
1	Dimethyl sulfide	C75183	C_2_H_6_S	62.1	789.3	238.388	0.964
2	Propanal (D)	C123386	C_3_H_6_O	58.1	833.3	261.118	1.148
3	Propanal (M)	C123386	C_3_H_6_O	58.1	834.0	261.519	1.046
4	2-Methyl propanal (M)	C78842	C_4_H_8_O	72.1	843.4	266.672	1.099
5	2-Methyl propanal (D)	C78842	C_4_H_8_O	72.1	845.3	267.734	1.282
6	Propan-2-one	C67641	C_3_H_6_O	58.1	847.2	268.736	1.117
7	Methyl acetate	C79209	C_3_H_6_O_2_	74.1	859.3	275.563	1.031
8	Tetrahydrofuran (D)	C109999	C_4_H_8_O	72.1	861.4	276.755	1.231
9	Tetrahydrofuran (M)	C109999	C_4_H_8_O	72.1	863.7	278.108	1.064
10	Ethyl acetate (M)	C141786	C_4_H_8_O_2_	88.1	900.8	300.318	1.099
11	Ethyl acetate (D)	C141786	C_4_H_8_O_2_	88.1	903.2	301.799	1.342
12	Butan-2-one	C78933	C_4_H_8_O	72.1	916.0	309.905	1.250
13	Methanol	C67561	CH_4_O	32.0	920.8	312.969	0.982
14	3-Methyl butanal	C590863	C_5_H_10_O	86.1	930.3	319.243	1.403
15	Ethanol	C64175	C_2_H_6_O	46.1	940.1	325.777	1.146
16	Pentanal	C110623	C_5_H_10_O	86.1	979.2	353.205	1.430
17	1-Penten-3-one	C1629589	C_5_H_8_O	84.1	1009.6	379.249	1.323
18	1-Propanol (D)	C71238	C_3_H_8_O	60.1	1050.5	423.267	1.255
19	1-Propanol (M)	C71238	C_3_H_8_O	60.1	1051.8	424.736	1.110
20	Camphene	C79925	C_10_H_16_	136.2	1076.7	453.960	1.218
21	Hexanal (M)	C66251	C_6_H_12_O	100.2	1097.2	479.693	1.257
22	Hexanal (D)	C66251	C_6_H_12_O	100.2	1098.7	481.988	1.567
23	2-Methyl-1-propanol (D)	C78831	C_4_H_10_O	74.1	1102.7	488.251	1.364
24	2-Methyl-1-propanol (M)	C78831	C_4_H_10_O	74.1	1106.3	493.883	1.167
25	Butan-1-ol (D)	C71363	C_4_H_10_O	74.1	1155.7	578.603	1.376
26	Butan-1-ol (M)	C71363	C_4_H_10_O	74.1	1156.1	579.398	1.186
27	1-Penten-3-ol	C616251	C_5_H_10_O	86.1	1168.5	602.797	0.945
28	Alpha-terpinene	C99865	C_10_H_16_	136.2	1173.5	612.495	1.218
29	Ethyl crotonate (D)	C623701	C_6_H_10_O_2_	114.1	1177.3	620.106	1.555
30	Ethyl crotonate (M)	C623701	C_6_H_10_O_2_	114.1	1178.7	622.787	1.181
31	Heptan-2-one	C110430	C_7_H_14_O	114.2	1186.0	637.530	1.265
32	Cyclopentanone	C120923	C_5_H_8_O	84.1	1191.4	648.753	1.106
33	Heptanal	C111717	C_7_H_14_O	114.2	1193.6	651.975	1.331
34	Limonene (M)	C138863	C_10_H_16_	136.2	1202.8	663.613	1.224
35	Limonene (D)	C138863	C_10_H_16_	136.2	1203.1	664.031	1.294
36	3-Methyl-1-butanol (M)	C123513	C_5_H_12_O	88.1	1216.8	681.825	1.246
37	3-Methyl-1-butanol (D)	C123513	C_5_H_12_O	88.1	1217.0	682.067	1.490
38	(E)-2-hexenal	C6728263	C_6_H_10_O	98.1	1226.2	694.257	1.183
39	Gamma-terpinene	C99854	C_10_H_16_	136.2	1250.1	727.017	1.220
40	1-Pentanol	C71410	C_5_H_12_O	88.1	1259.8	740.707	1.256
41	2-Methyl tetrahydrofuran-3-one (M)	C3188009	C_5_H_8_O_2_	100.1	1274.2	761.668	1.075
42	2-Methyl tetrahydrofuran-3-one (D)	C3188009	C_5_H_8_O_2_	100.1	1274.5	762.062	1.427
43	3-Hydroxybutan-2-one (M)	C513860	C_4_H_8_O_2_	88.1	1295.0	792.784	1.052
44	3-Hydroxybutan-2-one (D)	C513860	C_4_H_8_O_2_	88.1	1296.4	794.982	1.333
45	1-Hydroxypropan-2-one (M)	C116096	C_3_H_6_O_2_	74.1	1308.8	814.758	1.032
46	1-Hydroxypropan-2-one (D)	C116096	C_3_H_6_O_2_	74.1	1310.1	816.955	1.236
47	Ethyl lactate (D)	C97643	C_5_H_10_O_3_	118.1	1356.1	895.115	1.543
48	Ethyl lactate (M)	C97643	C_5_H_10_O_3_	118.1	1356.4	895.637	1.139
49	Nonanal	C124196	C_9_H_18_O	142.2	1405.1	986.519	1.474
50	2-Cyclohexen-1-one	C930687	C_6_H_8_O	96.1	1441.7	1060.988	1.110
51	Furfural (M)	C98011	C_5_H_4_O_2_	96.1	1486.9	1160.527	1.086
52	Furfural (D)	C98011	C_5_H_4_O_2_	96.1	1493.1	1174.958	1.345
53	Acetic acid	C64197	C_2_H_4_O_2_	60.1	1496.2	1182.140	1.159
54	2-Acetyl furan	C1192627	C_6_H_6_O_2_	110.1	1537.9	1284.113	1.112
55	Benzaldehyde (D)	C100527	C_7_H_6_O	106.1	1548.3	1310.942	1.474
56	Benzaldehyde (M)	C100527	C_7_H_6_O	106.1	1549.1	1313.162	1.152
57	Propanoic acid	C79094	C_3_H_6_O_2_	74.1	1631.7	1547.304	1.102
58	Isobutyric acid (D)	C79312	C_4_H_8_O_2_	88.1	1700.0	1771.927	1.368
59	Isobutyric acid (M)	C79312	C_4_H_8_O_2_	88.1	1701.7	1777.724	1.154
60	Gamma-butyrolactone (D)	C96480	C_4_H_6_O_2_	86.1	1703.8	1785.192	1.306
61	Gamma-butyrolactone (M)	C96480	C_4_H_6_O_2_	86.1	1707.6	1798.815	1.084

^a^RI represents the retention index calculated using an MXT-WAX metal capillary chromatographic column.^b^Rt represents the retention time in the capillary GC column.^c^Dt represents the drift time in the drift tube.^d^D, dimer; M, monomer.

This Maillard reaction, also known as a carbonyl–amino compound reaction, refers to the formation of a dark brown substance because of the rearrangement, dehydration, condensation, and polymerization of carbonyl and amino compounds. Rhubarb contains sugars, amino acids, and polyphenols, which provide conditions for enzymatic browning and facilitate the Maillard reaction during processing (26).

### 3.2 Complete spectral analysis of rhubarb samples

**Figure 2:**
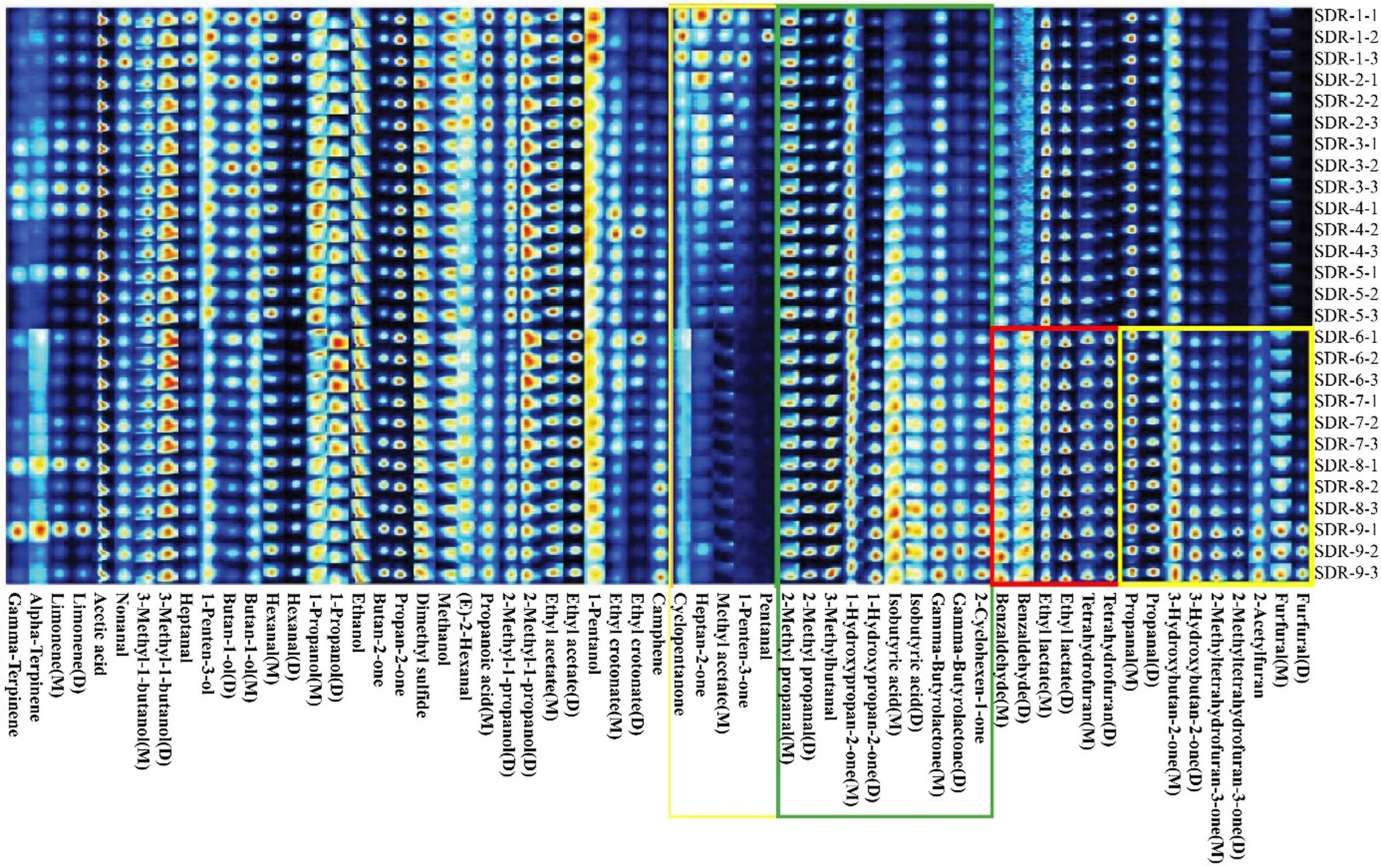
Gallery Plot maps (fingerprints) of the volatile organic compounds in rhubarb samples at different steaming and sun-drying times.

Complete data on VOCs and their differences among the rhubarb samples are shown in Figure 2. The contents in the areas denoted with yellow, red, and green rectangles significantly increased during steaming and sun-drying. Furfural, 2-acetyl furan, 2-methyl tetrahydrofuran-3-one, 3-hydroxy butan-2-one, and propanal contents in the yellow rectangle were higher in SDR-6–9 than in SDR-1–5, whereas it gradually increased in SDR-6–9. In the red rectangle, tetrahydrofuran, ethyl lactate, and benzaldehyde contents were higher in SDR-6–9 than in SDR-1–5, whereas those in SDR-6–9 were stable. The contents of 2-cyclohexen-1-one, gamma-butyrolactone, isobutyric acid, 1-hydroxy propan-2-one, 3-methyl butanal, and 2-methyl propanal in the green rectangle gradually increased during processing. However, the contents of pentanal, 1-penten-3-one, methyl acetate, heptan-2-one, and cyclopentanone in the orange rectangle significantly decreased during processing. Furthermore, the relative contents of other compounds fluctuated during processing without any significant differences.

### 3.3 Cluster analysis of characteristic volatile flavoring compounds in rhubarb samples

**Figure 3:**
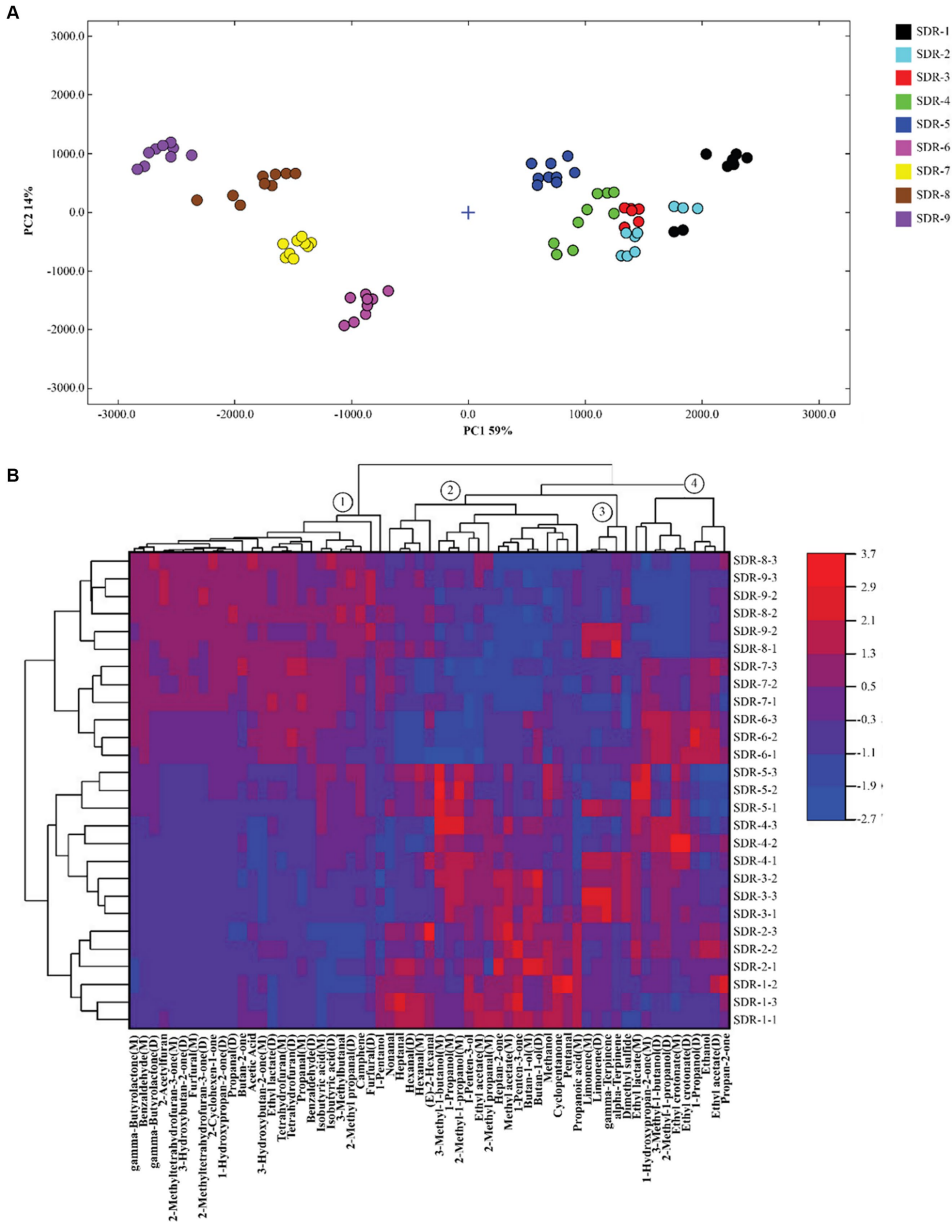
Principal component analysis **(A)** and cluster heat mapping **(B)** of the characteristic volatile components in rhubarb samples at different steaming and sun-drying times.

The differences in volatile compounds were analyzed using PCA. As shown in Figure 3A, the cumulative variance contribution rate of PC1 (14%) and PC2 (59%) was 73%, indicating that the PCA separation model was effective. The PCA plot indicated that the distance between SDR-1–5 and SDR-6–9 was relatively large, as evidenced by two distinct stages. The distance among SDR-1–5 was relatively close, except for SDR-1, and the differences between the groups were small. The distance interval among SDR-6–9 was similar, and they clustered with one another, indicating little difference in composition; however, each sample could be distinguished from the others.

To further assess the changes in the characteristic volatile components of rhubarb after different steaming and sun-drying times, cluster heat maps were generated based on the peak intensities of 61 characteristic markers (Figure 3B). The response value of the compound increased with increasing red intensity and decreased with increasing blue intensity. As shown in the figure, rhubarb processing can be divided into two stages: odor fluctuation and odor stability. In Figure 3B, these odor substances are divided into four categories according to the clustering results, namely, ①, ②, ③, and ④. The composition of the odor substances varied at different stages; among these, ① compounds played a crucial role in distinguishing the different stages. The odor fluctuation stage included SDR-1–5. At this stage, the response value of the ① compounds was low, indicating that no changes occurred in these odor substances during the first to fifth cycles of steaming and sun-drying. The odor-stable stage included SDR-6–9, which formed a clear boundary with the fifth cycle. The response value of ① compounds was significantly increased (*p* < 0.05) and reached the maximum value, reaching a stable stage of rhubarb odor. A total of 26 volatile odor substances, including the eight aldehydes propanal (M), propanal (D), benzaldehyde (M), benzaldehyde (D), furfural (M), furfural (D), 3-methyl butanal, and 2-methyl propanal (D) were present in the ① compounds. These usually have a low odor threshold and contribute significantly to odor. Furans, such as 2-acetyl furan, tetrahydrofuran (M), tetrahydrofuran (D), 2-methyl tetrahydrofuran-3-one (M), and 2-methyl tetrahydrofuran-3-one (D), are products of the Maillard reaction that occurs during the heat processing of rhubarb. They also have a low threshold and significantly contribute to odor. The Maillard reaction, also known as a carbonyl–amino compound reaction, refers to the formation of a dark brown substance through rearrangement, dehydration, condensation, and polymerization of carbonyl and amino compounds. Rhubarb contains sugars, amino acids, and polyphenols, which provide conditions for enzymatic browning and facilitate the Maillard reaction during processing (26). The proportion of these two types of compounds and their respective high response values are responsible for the primary odor of the SDR-9 samples. As a result, these eight aldehydes and five furans are key odor substances after nine cycles of steaming and sun-drying. Contrary to the ① compounds, ②, ③, and ④ compounds were present in high quantities in SDR-1–5. They exhibited a different pattern from the sixth round of steaming and sun-drying. The volatile components in the ② compounds, such as nonanal, heptanal, hexanal (M, D), 2-methyl propanol (M), (E)-2-hexanal, 3-methyl-1-butanol (M), 1-propanol, 2-methyl-1-propanol (M), 1-penten-3-ol, and ethyl acetate, significantly decreased (*p* < 0.05) after six to seven cycles of steaming and sun-drying. The other volatile substances in the ② compounds continued to decrease from the sixth to the ninth cycle. The ③ compounds primarily comprised four olefins and one sulfide, which fluctuated during the process. The ④ compounds were mainly alcohols, esters, and ketones, which reached their highest values during the sixth and seventh cycles drying, followed by a decrease in the eighth and ninth cycles.

Taken together, six steaming and sun-drying cycles may be considered a key process point. After six rounds, the rhubarb composition tends to be stable, and there is no significant difference in composition between SDR-6 and SDR-9. Therefore, replacing SDR-9 with SDR-6 is an effective method to streamline the process.

### 3.4 Quantitative analysis of anthraquinones using HPLC

#### 3.4.1 Separation of nine standard components using HPLC

**Figure 4:**
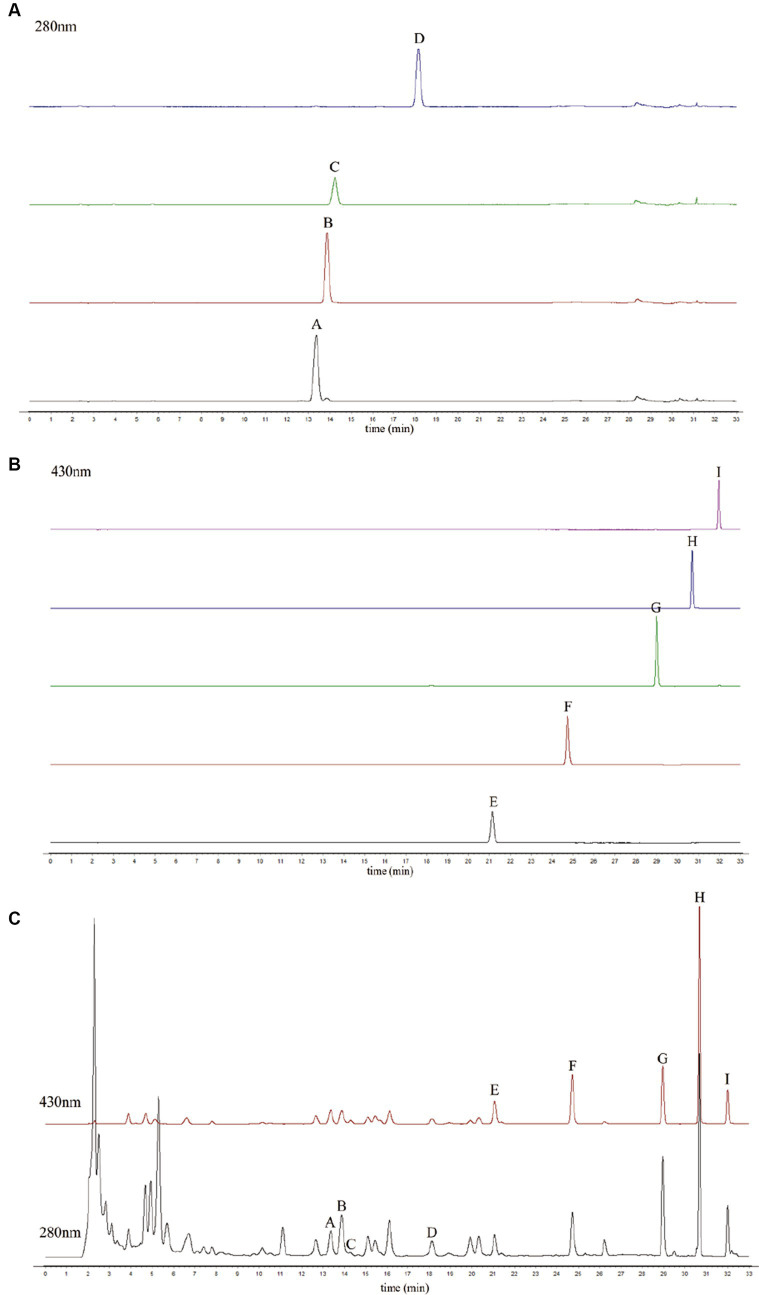
High-performance liquid chromatography (HPLC) chromatogram of the standard components at 280 nm **(A)**, at 430 nm **(B)**, and the raw rhubarb samples **(C)**. (A) Chrysophanol-8-O-β-D-glucoside, (B) emodin-8-O-β-D-glucoside, (C) aloe-emodin-3-(hydroxymethyl)-O-β-D-glucoside, (D) physcion-8-O-β-D-glucoside, (E) aloe-emodin,(F) rhein, (G) emodin, (H) chrysophanol, and **(I)** physcion.

The standard solution was separated by HPLC. The retention times of chrysophanol-8-O-β-D-glucoside, physcion-8-O-β-D-glucoside, aloe-emodin-3-(hydroxymethyl)-O-β-D-glucoside, emodin-8-O-β-D-glucoside, aloe-emodin, rhein, emodin, chrysophanol, and physcion were 13.60, 13.85, 14.27, 18.14, 21.07, 24.73, 28.86, 30.70, and 31.87 min, respectively (Figures 4A,B). This method was also applied to the prepared rhubarb samples (Figure 4C).

#### 3.4.2 Linearity, limit of detection, and limit of quantification

Linearity was defined using a calibration curve. Six different volumes (1, 5, 10, 15, 20, and 25 μL) of the standard solution were used to establish a calibration curve. The following linear regression equation was obtained from the calibration curve: *Y* = *a x* + *b*, where *a* is the slope and *b* is the intercept of the calibration curve, *x* is the infection volume of the standard components, and *Y* is the peak area. The correlation coefficients for all marker components exhibited excellent linearity (*R*^2^ > 0.9992). The linear relationship of components A–I was good at 0.0164–0.4100, 0.0201–0.5025, 0.0097–0.2425, 0.0142–0.3550, 0.0200–0.5000, 0.0270–0.6750, 0.0780–1.9500, 0.0344–0.8600, and 0.0222–0.5550 μg, respectively. The limit of detection (LOD) and limit of quantification (LOQ) were calculated based on the standard deviation (SD) and the slope (S) of the calibration curve using the equations: LOD = (3.3 × SD)/S and LOQ = (10 × SD)/S. From the calibration curve of the peak area versus concentration, the SD was the SD of the response. It was estimated by the SD of the intercepts of the regression line in the calibration curve. S was the slope of the calibration curve. Table 3 lists the specific results.

**Table 3:** Linearity, correlation coefficient (R2), limit of detection (LOD), and limit of quantification (LOQ) of the study compounds.

Components	Regression equation	*R*^2^ (*n* = 6)	LOD (μg/mL)	LOQ (μg/mL)
Chrysophanol-8-O-β-D-glucoside	*Y* = 1129835.5134 *x* − 4143.8306	0.9997	0.107	0.324
Emodin-8-O-β-D-glucoside	*Y* = 1166589.5924 *x* − 4843.5435	0.9999	0.051	0.153
Aloe-emodin-3-(hydroxymethyl)-O-β-D-glucoside	*Y* = 1566373.4619 *x* − 1584.9194	0.9999	0.014	0.043
Physcion-8-O-β-D-glucoside	*Y* = 646464.1072 *x* − 1080.1774	0.9999	0.068	0.207
Aloe-emodin	*Y* = 2655755.6048 *x* − 3898.7532	1.0000	0.032	0.098
Rhein	*Y* = 2383813.2019 *x* − 31300.4484	0.9998	0.001	0.003
Emodin	*Y* = 2411001.4165 *x* − 108200.5661	0.9995	0.069	0.208
Chrysophanol	*Y* = 1263925.5673 *x* − 25731.3339	0.9992	0.059	0.180
Physcion	*Y* = 1958043.5193 *x* − 9490.6710	0.9999	0.037	0.112

#### 3.4.3 Precision

The raw rhubarb solution was injected continuously six times based on the chromatographic conditions described in Section 2.2, with an injection volume of 10 μL. The repeatability of the analytical method was considered reliable according to the RSD (1.98 < 2%). The anthraquinone content in the raw rhubarb is listed in Table 4.

**Table 4:** Analytical results of the precision tests.

Components	Reference concentration (μg/mL)	Mean ± SD (%)	RSD (%)
Chrysophanol-8-O-β-D-glucoside	16.40	0.1304 ± 0.0007	0.52
Emodin-8-O-β-D-glucoside	20.10	0.1095 ± 0.0005	0.44
Aloe-emodin-3-(hydroxymethyl)-O-β-D-glucoside	9.70	0.0422 ± 0.0002	0.39
Physcion-8-O-β-D-glucoside	14.20	0.0449 ± 0.0006	1.24
Aloe-emodin	20.00	0.0707 ± 0.0013	1.90
Rhein	27.00	0.1419 ± 0.0028	1.98
Emodin	78.00	0.1681 ± 0.0028	1.65
Chrysophanol	34.40	0.3208 ± 0.0054	1.67
Physcion	22.20	0.1143 ± 0.0017	1.44

#### 3.4.4 Robustness

The rhubarb sample solution was injected at 0, 2, 4, 8, 12, and 24 h after preparation. Table 5 lists the robustness of the contents. An RSD value of less than 2% indicated that the sample composition was not changed significantly within 24 h; therefore, the method was considered robust.

**Table 5:** Analytical results of the robustness of the method.

Components	Mean ± SD of peak area	RSD (%)
Chrysophanol-8-O-β-D-glucoside	586,544 ± 3,944	0.67
Emodin-8-O-β-D-glucoside	975,405 ± 3,021	0.31
Aloe-emodin-3-(hydroxymethyl)-O-β-D-glucoside	76,240 ± 568	0.75
Physcion-8-O-β-D-glucoside	376,999 ± 1,105	0.29
Aloe-emodin	429,562 ± 8,048	1.87
Rhein	760,992 ± 11,225	1.48
Emodin	712,528 ± 8,988	1.26
Chrysophanol	1,886,078 ± 29,245	1.55
Physcion	364,090 ± 5,181	1.42

#### 3.4.5 Accuracy

The accuracy of the method was verified by a recovery test. The recovery test was done by adding a standard solution to a known quantity of rhubarb sample. The assay was repeated six times. The recovery values of the nine components varied between 97.48 and 104.99% and the RSD values were between 0.50 and 1.76% (Table 6).

**Table 6:** Analytical results of the recovery of the method.

Components	Theoretical yield (mg)	Actual yield (mg)	Recovery (%)	RSD (%)
Chrysophanol-8-O-β-D-glucoside	0.6041	0.6023	99.37	1.25
	0.6030	0.6019	99.61	
	0.6056	0.5986	97.48	
	0.6036	0.6072	101.29	
	0.6008	0.5974	98.77	
	0.5991	0.5969	99.19	
Emodin-8-O-β-D-glucoside	0.4750	0.4839	104.44	0.71
	0.4752	0.4852	104.97	
	0.4756	0.4817	103.05	
	0.4751	0.4832	104.05	
	0.4752	0.4847	104.73	
	0.4753	0.4853	104.99	
Aloe-emodin-3-(hydroxymethyl)-O-β-D-glucoside	0.2021	0.2043	102.28	1.76
	0.202	0.2029	100.91	
	0.2019	0.2037	101.86	
	0.202	0.2017	99.67	
	0.2021	0.2038	101.71	
	0.2024	0.2001	97.59	
Physcion-8-O-β-D-glucoside	0.3075	0.3101	101.83	1.40
	0.3074	0.3126	103.63	
	0.308	0.3141	104.30	
	0.3074	0.3086	100.86	
	0.3081	0.3144	104.44	
	0.3076	0.3111	102.46	
Aloe-emodin	0.3624	0.3652	101.93	0.69
	0.363	0.3660	102.11	
	0.3627	0.3673	103.23	
	0.3622	0.3650	101.96	
	0.3628	0.3676	103.31	
	0.3624	0.3648	101.68	
Rhein	0.7172	0.7212	101.48	0.98
	0.7161	0.7201	101.47	
	0.717	0.7243	102.69	
	0.7161	0.7271	104.09	
	0.7179	0.7229	101.85	
	0.7176	0.7227	101.87	
Emodin	0.7063	0.7182	104.06	0.51
	0.7065	0.7184	104.07	
	0.7058	0.7189	104.49	
	0.7061	0.7194	104.53	
	0.7065	0.7164	103.37	
	0.7075	0.7172	103.31	
Chrysophanol	1.4813	1.4985	102.53	0.80
	1.4743	1.4994	103.70	
	1.4756	1.5003	103.63	
	1.4759	1.4957	102.91	
	1.4305	1.4611	104.82	
	1.4305	1.4568	104.14	
Physcion	0.5074	0.5161	103.93	0.50
	0.5074	0.5146	103.24	
	0.5089	0.5146	102.56	
	0.5077	0.5142	102.93	
	0.5080	0.5141	102.77	
	0.5078	0.5135	102.59	

#### 3.4.6 Analysis of anthraquinone components in different rhubarb samples

The SDR-9 samples were analyzed by HPLC. The amount of the anthraquinone components was calculated from the calibration curve of the standards. Table 7 lists the content of the nine components in the nine samples.

**Table 7:** Components of nine anthraquinone components in the SDR samples.

	Content (mg/g, %)								
	A	B	C	D	E	F	G	H	I
SDR-1	96.4 ± 2.1	69.2 ± 2.4	51.1 ± 1.0	37.5 ± 2.7	86.1 ± 5.0	168.6 ± 10.8	184.6 ± 6.7	394.8 ± 25.2	140.6 ± 35.9
SDR-2	123.1 ± 5.4	99.6 ± 3.5	37.4 ± 1.7	50.2 ± 3.1	63.2 ± 3.1	121.7 ± 6.6	132.5 ± 4.7	282.0 ± 9.8	99.4 ± 22.9
SDR-3	128.8 ± 1.8	84.6 ± 1.7	47.7 ± 1.5	51.7 ± 2.8	67.3 ± 3.4	131.6 ± 5.2	142.9 ± 5.3	295.0 ± 10.8	127.8 ± 21.7
SDR-4	113.0 ± 8.0	74.4 ± 5.2	39.7 ± 3.6	46.0 ± 4.7	65.9 ± 5.7	111.6 ± 18.8	136.6 ± 10.0	267.2 ± 17.0	115.1 ± 23.7
SDR-5	90.8 ± 4.3	56.5 ± 2.5	38.8 ± 1.7	37.3 ± 2.2	62.2 ± 2.6	107.3 ± 6.8	125.5 ± 2.2	294.5 ± 10.7	122.4 ± 21.0
SDR-6	96.2 ± 1.0	58.6 ± 1.0	36.4 ± 1.2	35.1 ± 1.6	55.7 ± 1.5	107.7 ± 1.2	121.0 ± 2.5	241.7 ± 5.6	97.7 ± 15.8
SDR-7	69.4 ± 8.3	53.7 ± 6.2	33.5 ± 3.9	29.3 ± 2.3	54.9 ± 5.8	102.2 ± 12.4	119.4 ± 13.0	231.1 ± 20.3	97.6 ± 7.3
SDR-8	61.6 ± 7.8	39.6 ± 4.8	32.0 ± 3.7	25.4 ± 2.5	56.2 ± 5.7	100.4 ± 12.7	119.5 ± 12.5	231.5 ± 19.3	103.1 ± 12.3
SDR-9	71.1 ± 7.9	39.9 ± 4.4	33.5 ± 3.7	28.6 ± 3.1	56.9 ± 5.0	94.8 ± 10.5	112.5 ± 10.5	246.5 ± 17.5	104.3 ± 20.2

### 3.5 Taste analysis with an electronic tongue

**Figure 5:**
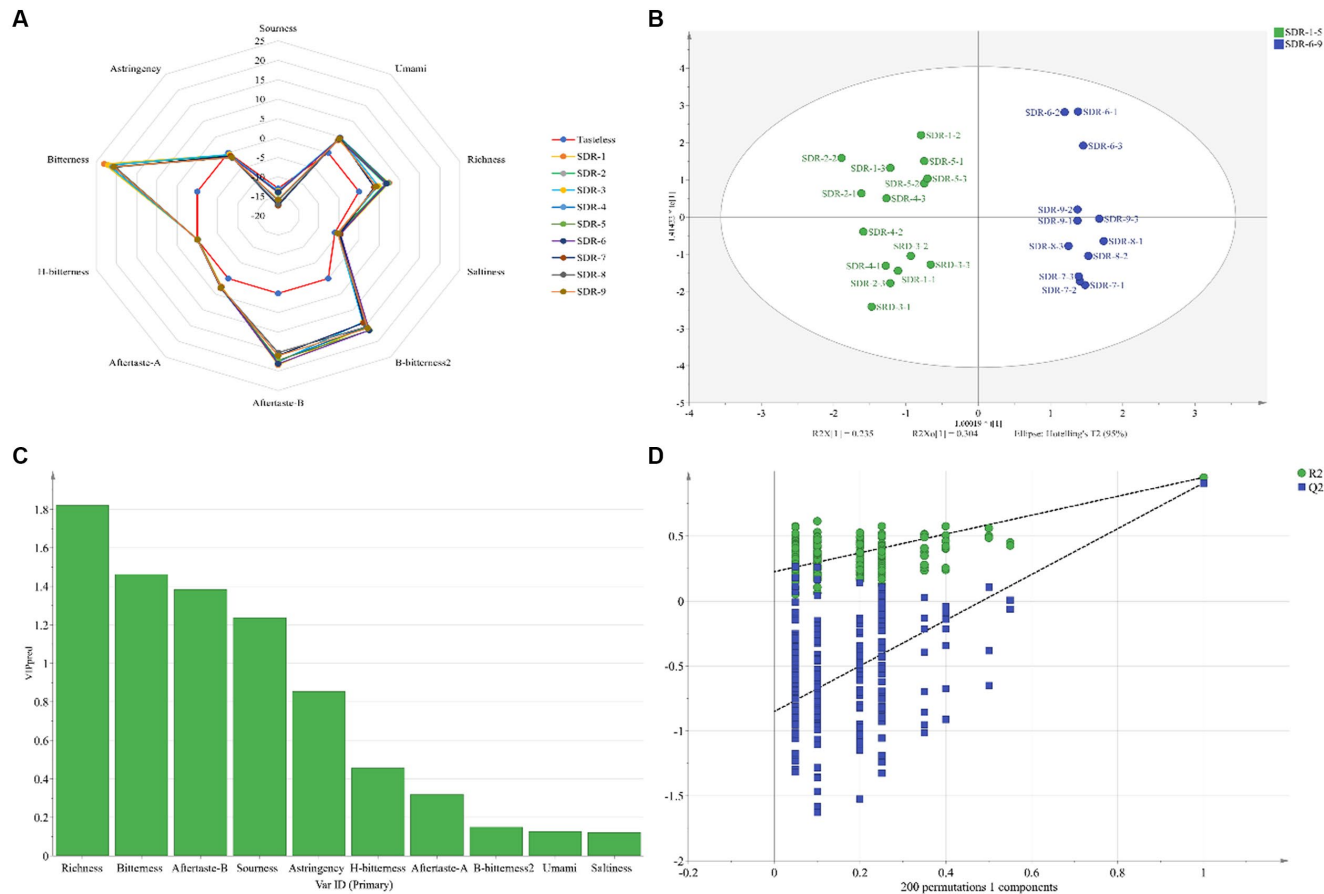
Odor composition **(A)**, score plots of the OPLS-DA model **(B)**, VIP value chart **(C)**, and permutation test plot **(D)** of rhubarb samples at different steaming and sun-drying times using an electronic tongue.

For the electronic tongue analysis, artificial saliva (also called reference solution) was used as the standard output. The state of the artificial saliva tested using the electronic tongue simulates the state of saliva in humans. The tasteless point is the output value of the reference solution. The acid tasteless point of the reference solution (reference) is negative 13, whereas the salty tasteless point is negative 6. When the taste value of the sample is lower than that of the tasteless point, the sample does not have taste, and vice versa. The richness in Table 2 represents the aftertaste of umami and reflects the persistence of the freshness of the sample. A bitter aftertaste (aftertaste-B) reflects the residual degree of bitterness, whereas an astringent aftertaste (aftertaste-A) reflects the residual degree of astringency. The prominent taste characteristics of the rhubarb samples included bitterness, aftertaste-B, B-bitterness2, richness, umami, and aftertaste-A (Figure 5A). The other taste response values were close to or below the tasteless point, and the differences between samples were primarily reflected in richness. Of these, bitterness had the highest response value and contributed the most to the taste of the rhubarb samples.

Orthogonal partial least squares discriminant analysis was used to analyze the data obtained from the electronic tongue. The score plot of the OPLS-DA model is shown in Figure 5B, in which *R*^2^*X*, *R*^2^*Y*, and *Q*^2^ were 0.956, 0.951, and 0.908, respectively. The values were all >0.5 and close to 1, indicating that the model had a high goodness-of-fit and prediction ability. The clustering results of SDR-6 and SDR-9 in the figure were close, indicating that their tastes were similar to one another. Rhubarb samples were distributed in different quadrants in Figure 5B, which was divided into two parts: SDR-1–5 and SDR-6–9. This indicates that their respective tastes are similar. The results were similar to those of the GC–IMS analysis. The variable influence on the projection (VIP) value chart (Figure 5C) reflects the contribution of the tastes to model classification, and VIP > 1 was considered the standard for screening the different tastes. Figure 5C shows that the tastes contributing mostly to the model classification were richness, bitterness, aftertaste-B, and sourness. In addition, the OPLS-DA model was verified using permutation tests (Figure 5D), which revealed that *R*^2^ and *Q*^2^ (*n* = 200) were 0.225 and − 0.851, respectively, indicating no over-fitting phenomenon in the reliability of the model.

### 3.6 Correlation analysis between the taste of rhubarb samples and content of nine anthraquinone components

**Figure 6:**
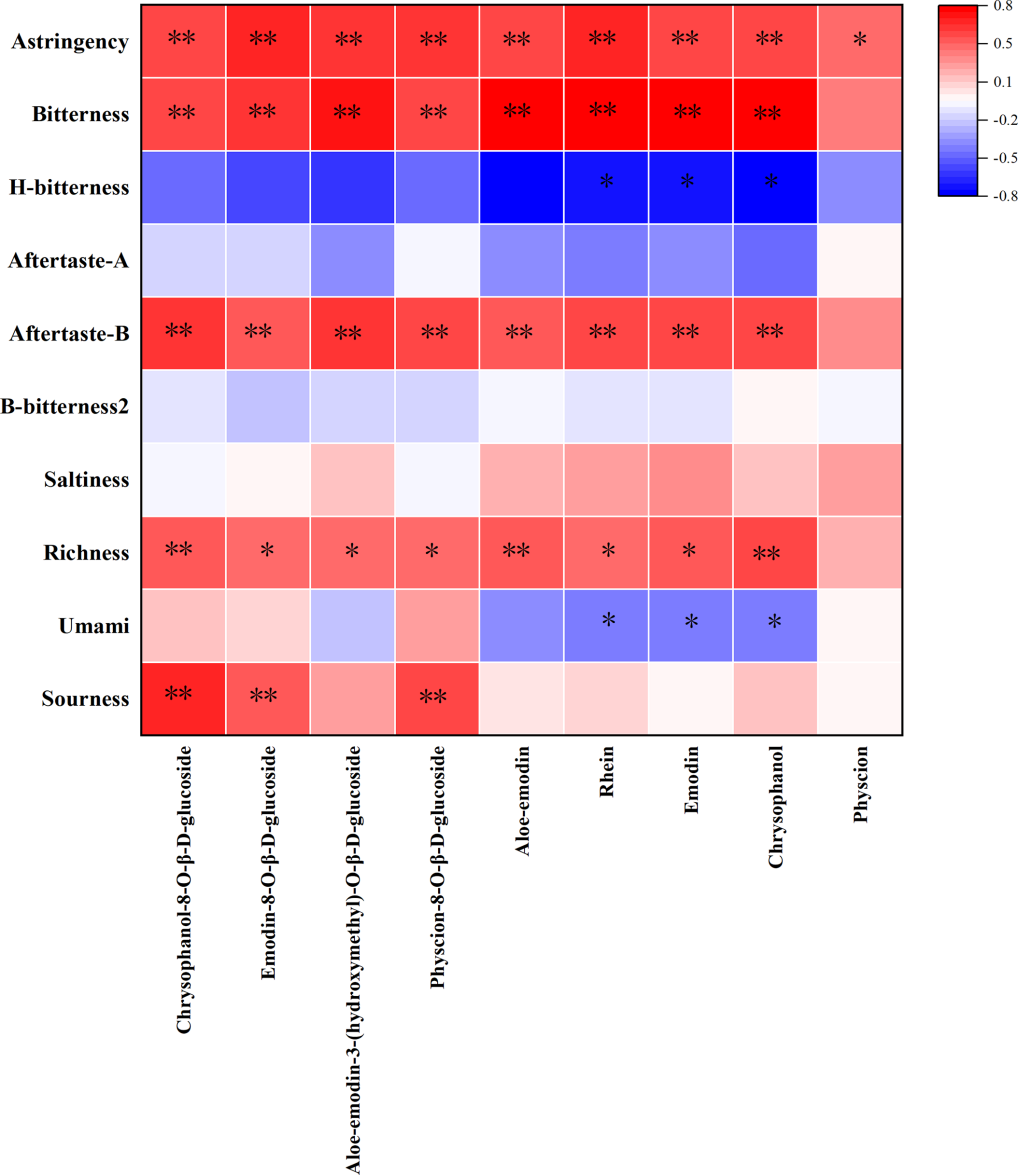
Heatmap of taste and anthraquinones.

Pearson correlation analysis was conducted using SPSS software (R26.0.0.0, IBM, United States) and the resulting data were imported into OriginPro 2021 software (v.9.8.0, OriginLab, Northampton, MA, United States) to generate a heatmap (Figure 6). The flavors with a significant positive correlation with anthraquinones was richness, aftertaste-B, bitterness, sourness, and astringency, which is similar to the results of the VIP value chart. Umami and H-bitterness were negatively correlated with the anthraquinone glycoside components.

## 4 Conclusion

Flavor analysis includes odor determination using GC–IMS and taste characterization using an electronic tongue. During the odor analysis of SDR-9, 61 volatile compounds, including aldehydes, esters, alcohols, ketones, acids, alkenes, and furans, were identified. Among these, 13 volatile components were the predominant substances for odor, which divided the process into two stages: 1–5 cycles of steaming and sun-drying and 6–9 times. In the second stage, SDR-6 and SDR-9 were grouped in terms of odor. The electronic tongue analysis revealed that the most prominent taste was bitterness. The OPLS-DA clustering results of SDR-6 and SDR-9 were close, indicating that their tastes are similar to one another. Moreover, anthraquinones were determined by HPLC and there was a significant correlation between taste and the components of the SDR samples. Based on the above results, there are similarities in smell, taste, and composition between SDR-6 and SDR-9; thus, the traditional process of steaming and sun-drying nine times can be reduced to six.

## Data availability statement

The original contributions presented in the study are included in the article; further inquiries can be directed to the corresponding author.

## Ethics statement

The animal study was approved by Experimental Animal Ethics Committee, Institute of Traditional Chinese Medicine, Chinese Academy of Traditional Chinese Medicine; Approval number 2023B109. The study was conducted in accordance with the local legislation and institutional requirements.

## Author contributions

TL: Data curation, Writing – original draft, Writing – review & editing. MY: Data curation, Formal analysis, Methodology, Writing – review & editing. YD: Conceptualization, Validation, Writing – review & editing. YX: Project administration, Supervision, Writing – review & editing. LL: Writing – review & editing, Data curation, Funding acquisition, Resources, Software.
